# Impaired NK and NKT cells immunobiology in rheumatoid arthritis

**DOI:** 10.1186/1479-5876-10-S3-P32

**Published:** 2012-11-28

**Authors:** Ashish Aggarwal, Aman Sharma, Archana Bhatnagar

**Affiliations:** 1 Dept. of Biochemistry, Panjab University, Chandigarh, India; 2 Dept. of Internal Medicine, Postgraduate Institute of Medical Education and Research, Chandigarh, India

## Introduction

Rheumatoid Arthritis (RA) is an autoimmune disease with uncertain pathophysiology involving many interwoven signalling cascades. ROS, NK and NKT cells might be crucial in the disease severity of RA.

However, the role of oxidative stress, its impact on NK and NKT cell immunobiology and disease activity (DAS28) is largely unknown.

## Aim

In this study, we assessed the role of oxidative stress and NK cell subsets in the disease pathogenesis of RA.

## Patients and methods

The study included 30 patients of RA attending the out-patient Dept. of Internal medicine, PGIMER Chandigarh. An equal number of age and sex matched healthy volunteers were included. The state of oxidative stress in various peripheral blood fractions, percentage NK and NKT cell expression, their altered apoptotic signaling pathways involving mitochondrial membrane potential, FAS associated death domain (FADD) mediated pathways and DNA damage were studied.

## Results

Results explained a state of profound oxidative stress in the peripheral blood of RA patients. The percentage NK and NKT cell subsets were found to be diminished while ROS levels were increased. The depolarized mitochondrial membrane potential, FAS, FASL and active caspase-3 positive NK and NKT cell subsets were much elevated in patients. The DNA damage, assessed as percentage of DNA in comet tail, was significantly elevated.

## Conclusions

The present study strongly supports the protective role of NK cell subsets in the pathogenesis of RA. Findings of the present work indicate increased cellular apoptosis of peripheral NK and NKT cells in the diseased condition. PBMC and RBC are the major sites of enhanced oxidative stress. The state of oxidative stress and altered immunobiology of NK and NKT cells were strongly correlated with DAS28 score.

